# Ku protein as a potential human T-cell leukemia virus type 1 (HTLV-1) Tax target in clastogenic chromosomal instability of mammalian cells

**DOI:** 10.1186/1742-4690-2-45

**Published:** 2005-07-13

**Authors:** Franca Majone, Roberto Luisetto, Daniela Zamboni, Yoichi Iwanaga, Kuan-Teh Jeang

**Affiliations:** 1 Department of Biology, University of Padua, Padua, Italy; 2 Laboratory of Molecular Microbiology, NIAID, NIH, Bethesda, Maryland, 20892-0460, USA

## Abstract

The HTLV-1 Tax oncoprotein rapidly induces cytogenetic damage which can be measured by a significant increase in the number of micronuclei (MN) in cells. Tax is thought to have both aneuploidogenic and clastogenic effects. To examine the cellular target for Tax which might mechanistically explain the clastogenic phenomenon, we tested the ability of Tax to induce MN in rodents cells genetically defective for either the Ku80 protein or the catalytic subunit of DNA protein kinase (DNAPKcs). We found that cells genetically mutated in Ku80 were refractory to Tax's induction of MN while cells knocked-out for DNAPKcs showed increased number of Tax-induced MN. Using a cytogenetic method termed FISHI (Fluorescent In Situ Hybridization and Incorporation) which measures the number of DNA-breaks in cells that contained unprotected 3'-OH ends, we observed that Tax increased the prevalence of unprotected DNA breaks in Ku80-intact cells, but not in Ku80-mutated cells. Taken together, our findings suggest Ku80 as a cellular factor targeted by Tax in engendering clastogenic DNA damage.

## Background

We previously demonstrated that expression of the HTLV-I Tax oncoprotein rapidly induces cytogenetic damage which is reflected in a significant increase in the prevalence of micronuclei (MN) in cells [1-4]. To further characterize the phenomenon of Tax associated clastogenic DNA-damage, we wished to examine the status DNA-breaks in the nucleus and in MN in the presence or absence of Tax [4]. Using a cytogenetic method termed FISHI (Fluorescent In Situ Hybridization and Incorporation), DNA-breaks in the nucleus and in MN with centric or acentric DNA fragments could be characterized for the presence or absence of free 3'-OH ends. In our definition, free 3'-OH ends represent breaks which are accessible to the *in situ *addition of digoxigenin (DIG) -labeled dUTP using terminal deoxynucleotidyl transferase. On the other hand, an absence of accessible 3'-OH ends suggests that the breaks are protected and masked by a protein complex. *In vivo*, unprotected free 3'-OH ends may progress to larger lesions leading to increasingly serious chromosomal lesions which may eventually sow the seed for cellular transformation.

In earlier studies, we had observed that Tax increased the frequency of MN containing centric DNA fragments with unprotected free 3'-OH ends and that Tax decreased the frequency of MN containing DNA fragments with incorporation-inaccessible (i.e. protected) 3'-OH ends. Based on an increase in free 3'-OH containing ends/breaks, we hypothesized that Tax interfered with a protective cellular mechanism(s) that may normally recruit a protein complex to newly created DNA breaks. Subsequent to the publication of our report [4], Gabet et al. [5] showed that in some settings Tax can repress the expression of the catalytic subunit of human telomerase (hTert).

Telomerase is a ubiquitously-expressed multi-protein complex composed of a catalytic subunit (hTert), two associated proteins (TP-1 and HSP 90), and a highly conserved RNA (hTR) component of ~400 nucleotides. hTert acts as a reverse transcriptase, and normally catalyses the addition of short repetitive sequences to the ends of chromosomes using an RNA-template embedded within the hTert holoenzyme. Telomerase is expressed in proliferating stem cells, in germ cells, in activated lymphocytes and in many neoplastic cells such as gastric and colorectal carcinoma, breast tumours and adrenal tumours [6,7], and in some pre-neoplastic growths [8]. It is generally assumed that telomerase is silent in most primary somatic cells. Interestingly, because of the manner by which eukaryotic cells replicate DNA, when a cell does not have active telomerase, telomeres at the ends of chromosomes shorten progressively after every cellular division. Once the telomeric repeats have reached a critically abbreviated state, further cell division cannot ensue. This constraint may explain the senescence seen for normal somatic cells.

Telomeric repeats at the ends of chromosomes also appear to serve an end-protective function. Chromosomal ends which lack telomeric repeats are labile for end-to-end chromosome fusion and exonucleolytic degradation which can progress to further genetic rearrangements/damages. Provocatively, such gross rearrangements/damages can, at a low frequency, fortuitously alter the genome in a way to actually induce telomerase activity in the genetically altered cells. Once induced, such telomerase activity could endow the cells with the capacity to proliferate indefinitely, and this event could represent a first step towards malignant transformation [9].

We previously hypothesized [4] that proteins such as Ku, Sir, and the DNA protein kinase catalytic subunit (DNAPKcs) which are normally found at telomeric ends of chromosomes could be recruited rapidly to *de novo *interstitial chromosomal breaks. We had proposed that *de novo *interstitial breaks may be recognized by hTert and be stabilized by the transient addition of telomeric repeats which could then recruit Ku, Sir and DNAPKcs proteins [4]. Of note, Ku and DNAPKcs are also components of the non-homologous end-joining (NHEJ) DNA repair pathway. NHEJ is important for the repair of double-stranded DNA breaks. Knock-out mice and cultured cells deficient for one or more components of the Ku-DNAPKcs complex show genome instability phenotypes [10-16].

Because Tax interferes with the stability of *de novo *DNA breaks [4] and because Ku and DNAPKcs proteins apparently contribute protection to DNA breaks, we wish to understand how Tax influences double stranded DNA-breaks in cells (e.g. hamster xrs-6 cells) which are either genetically mutated for the Ku80 protein [10,11] or knocked out for the DNAPKcs gene (e.g. mouse embryo DNAPKcs -/- fibroblasts) [12]. We reasoned that if Tax acts to subvert the Ku protein, then cells (i.e. xrs-6) already lost for Ku80 would not incur increased DNA-break instability when Tax is over-expressed. On the other hand, if Tax targets DNAPKcs function, then we would expect that DNAPKcs-/- cells would not show enhanced frequency of cytogenetic damage when Tax is over-expressed, while xrs-6 cells would. Here, we used xrs-6 cells, DNAPKcs-/- cells, and the technique of *in situ *DIG-dUTP incorporation to distinguish between Ku80 and DNAPKcs as a DNA-break stabilizing factor targeted by Tax.

## Results

### MN induction by Tax in hamster and mouse cells

Clastogenic and aneuploidogenic agents increase the frequency of micronuclei (MN) because they disturb genome stability control mechanisms [1,4]. The frequency of MN can be viewed as being proportional to the cell's (in)efficiency at maintaining its genomic integrity. The NHEJ (Non-Homologous End Joining) pathway is one of the major pathways which eukaryotes use to repair double-stranded DNA breaks. Ku and DNAPKcs subunits are important NHEJ protein components.

**Figure 1:**
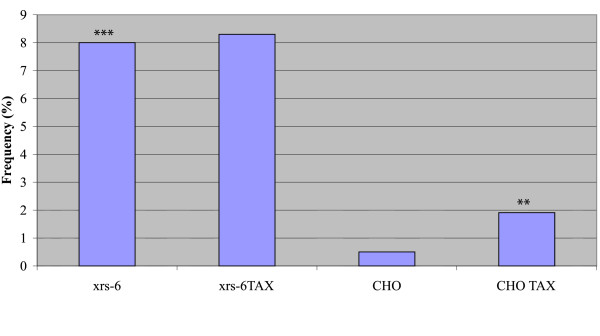
Frequency (%) of micronuclei containing cells in xrs-6 and CHO cell cultures without or with transfection by Tax. *** indicates significantly different value (P < 0.001, G test) from that found in CHO cells. ** indicates significantly different value (P < 0.01, G test) from that found in CHO cells.

**Figure 2:**
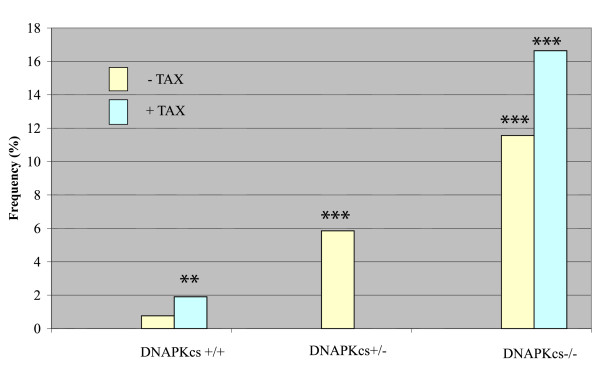
Frequency (%) of micronuclei in primary cultures of mouse embryo fibroblasts with indicated genotypes of DNAPKcs +/+, DNAPKcs +/-, or DNAPKcs -/- assayed without or with transfection of a Tax plasmid. *** indicates significantly different value (P < 0.001, G test) from that in DNAPKcs +/+ cells, with or without transfect with Tax plasmid. ** indicates significantly different value (P < 0.01, G test) from that in DNAPKcs +/+ cells without transfection with Tax plasmid.

To check Tax's effect in cells impaired for NHEJ, we first monitored the ambient frequency of micronuclei in hamster xrs-6 cells which have a mutated Ku80 gene [10,17]. We observed that MN frequency was significantly higher in xrs-6, than control CHO (Chinese hamster ovary) cells (Fig. 1). To the extent that MN reflects DNA-damage, this result suggests that under normal tissue culture conditions xrs-6 cells have a higher proclivity for cytogenetic damage. We next investigated mouse embryo fibroblasts (MEFs) engineered to be DNA-PKcs-/- [12]. We found that DNAPKcs-/- cells had a ten fold higher ambient frequency of MN when compared to wild type MEFs (DNA-PKcs+/+); and we also saw that DNAPKcs heterozygous MEFs (DNA-PKcs+/-) showed a five fold increase in MN over control MEFs (Fig. 2). Taken together, the results in figures 1 and 2 argue that both DNAPKcs and Ku proteins are important for the normal genomic homeostasis that prevents MN. Inactivation of either of these two NHEJ components appears to predispose the cell to increased cytogenetic damage.

We next compared MN frequencies in CHO, xrs-6, DNAPKcs+/+ and DNAPKcs-/- cells after transfection with a Tax-expression plasmid. Interestingly, after Tax transfection, the frequency of micronuclei in the xrs-6 cells did not significantly change from that seen in the same cells without Tax (Fig. 1). By contrast, Tax-transfected CHO cells showed a three fold increase in MN compared to mock transfected cells (Fig. 1). When we checked DNAPKcs+/+ and DNAPKcs-/- cells, we also found that both cell types showed increases in micronuclei after Tax-expression (Fig. 2).

We interpret the above results to mean that in Ku-intact cells (i.e. DNAPKcs+/+, DNAPKcs-/-, and CHO cells), Tax can increase cytogenetic damage above ambient levels. By contrast, Tax does not increase the extent of genetic damage in Ku defective cells (i.e. xrs-6 cells) (Fig. 1, 2). The two findings can be explained if Ku80 is specifically targeted by Tax. If so, because xrs-6 cells are already lost for Ku80, its already high baseline level of MN cannot be further aggravated by Tax. On the other hand, Tax could target the still intact Ku function in DNAPKcs+/+, DNAPKcs-/-, and CHO cells to increase MN numbers.

### DIG(digoxigenin)-dUTP incorporation in nuclei and MN of hamster and mouse cells

We next investigated the status of DNA breaks in the nuclei and MN of xrs-6, DNA-PKcs-/- and control cells using the previously described *in situ *DIG-dUTP incorporation assay [4]. This method incorporates *in situ *a tagged-dUTP which can be used to identify and quantify broken and unprotected 3'-OH DNA ends. We were curious to compare how Tax affects the protection of 3'-OH DNA ends in Ku80-/- (i.e. xrs-6) and DNAPKcs-/- cells.

**Figure 3:**
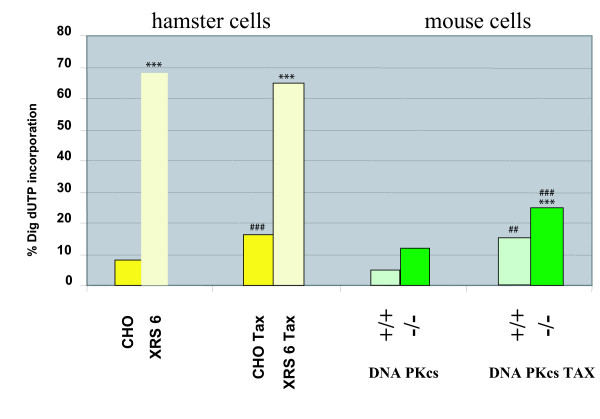
Comparison of the frequency of *in situ *incorporation of digoxigenin (DIG)-dUTP in nuclei of hamster and mouse cells in the absence or presence of Tax. *** indicates significantly different value (P < 0.001, G test) from that found in the respective control (comparison between the paired columns). ## or ### indicates significantly different value (P < 0.01, or P < 0.001, G test) from that of the respective controls in the absence of Tax.

**Figure 4:**
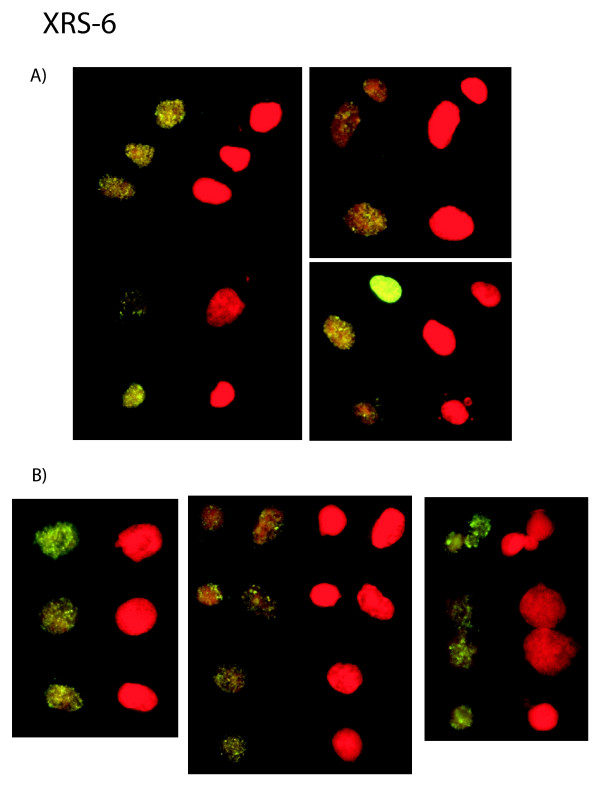
**Visualization of in situ incorporation of DIG-dUTP in xrs-6 cells in the absence (A) or presence (B) of transfected Tax. **Counterstaining with propidium iodide is shown as red fluorescence while incorporation of DIG-dUTP is shown as yellow-green fluorescence. Multiple views show that in situ incorporation signals in nuclei and micronuclei do not increase substantially after transfection with a Tax-expressing plasmid.

We found that the frequency of incorporated DIG-dUTP in nuclei and MN was significantly increased in xrs-6 cells compared to control CHO cells (Fig. 3). Under normal culturing conditions, xrs-6 cells showed robust and numerous *in situ *DIG-dUTP signals in nuclei and MN (Fig. 4A). These findings suggest that loss of Ku-function significantly increases the prevalence of unprotected freely accessible 3'-OH DNA ends. Interestingly, when we transfected Tax into xrs-6 cells, no further increase in DIG-dUTP incorporation in either the nuclei or MN was apparent (Fig. 4B). Thus, Tax expression in cells already lost for Ku80 failed to change further the number of unprotected 3'OH-DNA ends.

**Figure 5:**
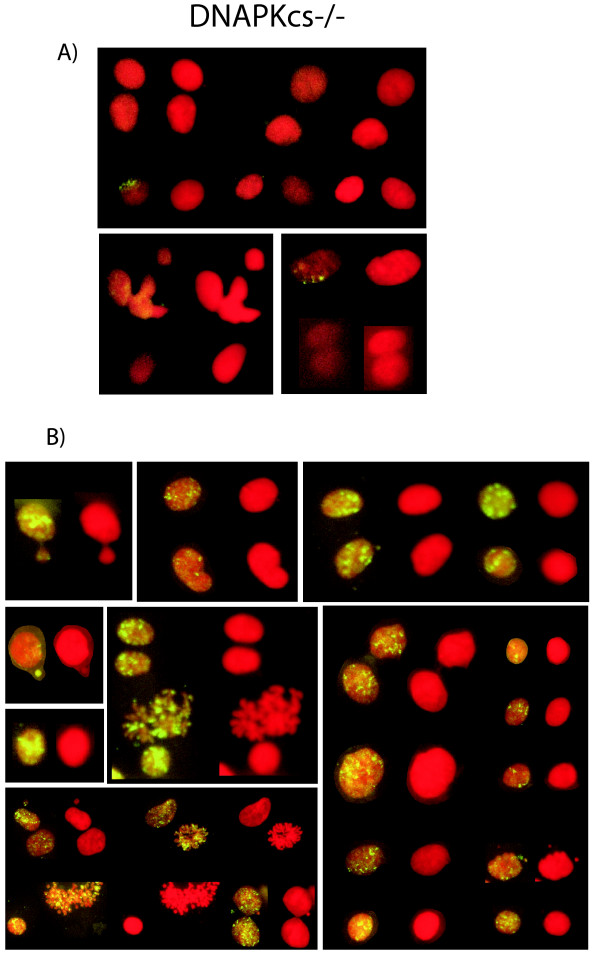
**Visualization of in situ incorporation of DIG-dUTP in PKcs-/- cells in the absence (A) or presence (B) of transfected Tax plasmid. **Counterstaining with propidium iodide is shown as red fluorescence while incorporation of DIG-dUTP is shown as yellow-green fluorescence. Multiple views show that in the presence of the Tax (B) the incorporation signals are far greater than those in the absence of Tax (A). Note that many MN are seen to contain in situ incorporation signals.

We also checked DNAPKcs-/- MEFs. These cells are knocked out for the DNAPKcs gene but have intact Ku80 protein. Here, we found that the ambient incorporation of DIG-dUTP into DNAPKcs-/- nuclei and MN was low (Fig. 3; Fig. 5A). Indeed, the DIG-dUTP incorporation frequency in DNAPKcs-/- cells was not significantly different from that in control DNAPKcs+/+ or in DNAPKcs+/- heterozygote cells (Fig. 3). After transfection with a Tax-plasmid, both DNAPKcs +/+ (Fig. 3) and DNA PKcs-/- (Fig. 3; Fig. 5B) showed significant increases in the incorporation of DIG-dUTP into nuclei and MN. Unlike xrs-6 cells, DNAPKcs-/- and DNAPKcs+/+ cells have intact Ku80; we interpret their DIG-dUTP incorporation results to mean that Tax targeted the Ku80 protein in these cells and that such targeting increased the number of DIG-dUTP accessible unprotected 3'OH DNA ends.

### Reduced Ku80 expression in HTLV-1 transformed cells

**Figure 6:**
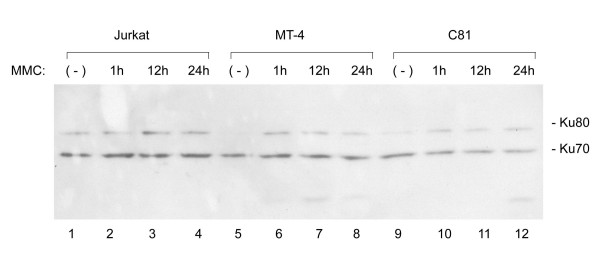
**Reduced constitutive expression of Ku80 in MT-4 and C81-6645 (C81) cells compared to Jurkat cells. **Total cell lysates were prepared from the indicated cell lines and probed with anti-serum which recognizes both Ku70 and 80 proteins. Where indicated the cells were also treated with 1 μM mitomycin C (MMC) for the stated time period before harvesting. Note that constitutively reduced Ku80 expression remains inducible by MMC in the two Tax expressing cell lines (MT-4 and C81).

The above findings suggested Ku80 as a Tax-target. To ask if Tax affects Ku80 in HTLV-1 transformed human cells, we investigated the expression of this protein in Jurkat, MT-4, and C81 cells (Fig. 6). Jurkat is a spontaneously transformed T-cell line unrelated to HTLV-1; while both MT-4 and C81 cells are HTLV-1 transformed cells that highly express Tax. Using anti-Ku antibody which recognizes both the Ku70 and Ku80 proteins, we found that constitutive expression of Ku80 was reduced in both cells that express Tax, MT-4 and C81 (Fig. 6, lanes 5 and 9), when compared to Jurkat (Fig. 6, lane 1). Interestingly, when cells were treated with mitomycin C (a DNA-damaging agent), Ku80 expression remained inducible in both MT-4 and C81 cells. Thus targeting of Ku80 by Tax appears not to be an irreversible process.

## Discussion

Tax has been reported to cause both aneuploidogenic and clastogenic effects. Here we have explored the clastogenic property of Tax. We posed a simple question: in cells respectively defective for either Ku80 or DNAPKcs, which cell type remains responsive to Tax-induction of MN and DIG-dUTP incorporation? Based on our results that cells genetically mutated in Ku80 were no longer responsive to Tax's induction of MN and DIG-dUTP incorporation, we posit that Ku80, but not DNAPKcs, is a functional Tax target.

Both Ku and DNAPKcs are important for NHEJ. The current thinking is that Ku protein binds to DNA discontinuously and in a sequence independent manner, carrying out a DNA-protective role [18]. Once bound to DNA, Ku proteins recruit and activate the catalytic DNAPKcs subunit which can phosphorylate Ku and other neighboring DNA-bound proteins [19]. It has also been reported that DNAPKcs self-phosphorylates to inactivate the holo-kinase complex and then dissociates itself from Ku and the DNA. In this manner, the helicase activity of Ku is inactivated, allowing base pairing to occur between micro-homologous regions. DNAPKcs further recruits the XRCC4/ligase IV protein, which provides the DNA-ligase function needed to complete repair [20]. This intimate interplay between DNAPKcs and Ku explains why an absence of one or the other protein results in increased cytogenetic aberrations in cells.

Ku and DNAPKcs are commonly found at the telomeric ends of chromosomes. One view is that these proteins with other factors assemble a telomeric "cap" which contributes to the stability of chromosome ends [21]. Of note, there is evidence which suggests that telomeric repeats may also be transiently added to *de novo *interstitial chromosomal breaks leading to their stabilization and preventing further exacerbation of damage [22]. Accordingly, DNA-ends or DNA-breaks not capped by telomeric sequences and their associated proteins are unstable and labile to aberrant fusions [23,13]. Interestingly, studies have shown that upon DNA damage, PARP-1 (a nuclear enzyme which catalyzes the polyADP-ribosylation of target proteins in response to DNA damage) and Ku proteins are rapidly activated and compete for binding to DNA-ends [24], suggesting a general activity conferred by these proteins in stabilizing damaged DNA [25]. PARP-1 and Ku proteins can be co-immunoprecipitated [26], indicating that the two DNA end-sensing molecules interact in response to DNA strand breakages. Moreover, Ku function can be modulated by PARP-1 [27,28]. Thus, PARP-1 polyADP-ribosylates itself and also Ku70/80, and the polyADP-ribosylated Ku 70/80 is reduced in its DNA binding affinity, and becomes attenuated in its ability to stimulate Werner syndrome (WRN) exonuclease [28].

Our current data add the viral Tax oncoprotein to the list of complex interactors with Ku. We report here that cells genetically knocked out for Ku80 are refractory to the induction by Tax of MN and DIG-dUTP incorporation. Interestingly, in cells intact for Ku80, Tax expression reduced the ambient expression of this protein. It remains to be resolved how Tax mechanistically affects Ku80-expression; however, adding our current to our previous demonstration that Tax interferes with the protective cellular mechanisms used normally for stabilizing DNA breaks [4,29], we propose that Ku80 likely represents a crucial DNA end-protective protein targeted by Tax. Targeting of DNA end-protective proteins by oncoproteins may attenuate the functions of these factors and could lead to increased DNA structural instability and progression of damage. Progression of DNA structural damage may ultimately contribute to and mechanistically explain the process of cellular transformation. Our views on the implications of protecting *de novo *DNA-breaks with telomeric-caps for cellular transformation are in part consistent with recent findings that telomeric fusion to breaks reduces oncogenic translocations and tumor formation [30].

## Materials and methods

### Cells and transfection

Hamster xrs-6 (genetically mutated for Ku 80) cells, CHO wild type cells, mouse embryo fibroblasts knocked out for the PKcs gene, and PKcs +/- or PKcs+/+ MEFs, were cultured as monolayers in Dulbecco's minimal essential medium (Invitrogen) supplemented with 10% fetal calf serum. Where indicated, cells were transiently transfected using calcium phosphate with a wild-type Tax expression plasmid (HPx). The cells were surveyed 48 hours later for cytogenetic effects.

### Micronuclei (MN) assay

For MN assay, suspensions of cells were prepared by trypsinization of cultured cells in log-phase. Cells were divided into 40 mm dishes with each dish receiving 8 × 10 ^5 ^cells in 10 ml of medium. The cells were collected 48 h later by trypsinization and were washed in phosphate-buffered saline and fixed for 15 minutes in paraformaldehyde (1% in PBS) for *in situ *incorporation analysis. Interphase preparations were obtained following the procedures previously described [1].

### Fluorescence *in situ *incorporation

Fluorescence *in situ *incorporation was carried out using terminal transferase (TdT) which catalyses the addition of deoxyribonucleotide triphosphates to the 3'-OH ends of single or double-stranded DNA. To the substrates of TdT, digoxigenin-11-dUTP (the digoxigenin is bound to position 5 of the pyrimidine by an arm of 11 carbon atoms) was added to the 3'-OH ends. Antibody detection of DIG-dUTP labelling employed a specific antibody linked to fluoresceine, a fluorochrome which when stimulated at 494 nm wavelength emits a green signal (λ = 523 nm). The experimental protocol for fluorescent *in situ *incorporation used 2 washes with HBS (NaCl 280 mM, Na_2_PO_4 _× 7H_2_O, 1.5 mM, Hepes 50 mM). The TdT incorporation reaction of DIG-11-dUTP used the following: 10 μl of a solution (Boheringer) containing potassium cocodylate 1 M, Tri-HCl 125 mM (pH 6.6, 4°C), Bovine serum albumin (BSA) 1.25 mg/ml, CoCl2 10 mM; 0.2 μl of a solution (Boheringer) containing TdT (25 units/μl), EDTA 1 mM, 2 mercaptoethanol 4 mM, glycerol 50% (v/v) (pH 6.6, 4°C); 1 μl of DIG-11-dUTP (1 mM) mixture (Boheringer). Distilled water was added to a final volume of 50 μl. The cells were incubated in this solution at 37°C for 1 hour in an HBS-moist environment. At the end of the incubation the slides were immersed into a basin containing 0.1% Triton X-100 and 0.5% BSA in HBS to equilibrate the slides with anti-DIG-11-dUTP (1:50) labelled with FITC (Boheringer). Equilibration was conducted at room temperature for 30 minutes in an HBS moist environment. The slides were subsequently washed 3 times for 5 minutes each with the same HBS solution. The slides were then counterstained with propidium iodide (0.3 μg/ml).

### Scoring of the slides

Fluorescent microscopy was performed on a Zeiss microscope with different filters and equipped with an HBO 100 mercury lamp (Osram, Munchen, Germany). Photographs were taken on Kodak Ektachrome 166 ASA film. To determine the number of MN per nucleus in slides, for each experimental point, 3000 cells were counted, using at least two independent slides for each experimental point. Differences between data from spontaneous and Tax induced cytogenetic effects were tested for significance using the G test [31].

## Competing interests

The author(s) declare that they have no competing interests.
